# Engagement with a Text-Based, Bilingual Blood Pressure Monitoring Program during Postpartum among a Multiethnic Population

**DOI:** 10.1097/NMC.0000000000001156

**Published:** 2025-12-03

**Authors:** Sarah Y. Nowlin, Natalie Boychuk, Nicole Essein, Kimberly Glazer, Frances M. Howell, Micki Burdick, Oluwadamilola Oshewa, Maria Monterroso, Alva Rodriguez, Camila Cabrera, Sheela Maru, Jennifer Lewey, Elizabeth A. Howell, Lisa Levine, Teresa Janevic

**Affiliations:** **Sarah Y. Nowlin** is an Assistant Professor in the Department of Population Health Science and Policy at Icahn School of Medicine at Mount Sinai Mount Sinai Hospital, Center for Nursing Research and Innovation, New York, NY. Dr. Nowlin can be reached at Sarah.nowlin@mountsinai.org; **Natalie Boychuk** is a Statistician in the Department of Epidemiology at Columbia University Mailman School of Public Health, New York, NY. The author can be reached at nab2194@cumc.columbia.edu; **Nicole Essein** is a Research Nurse in Lienhard School of Nursing at Pace University, Pleasantville, NY. The author can be reached at nicoleessien@gmail.com; **Kimberly Glazer** is an Assistant Professor in the Department of Population Health Science & Policy at Icahn School of Medicine at Mount Sinai, and Department of Obstetrics, Gynecology, and Reproductive Science at Icahn School of Medicine at Mount Sinai, New York, NY. Dr. Glazer can be reached at Kimberly.glazer@mountsinai.org; **Frances M. Howell** is a Clinical Research Coordinator in the Department of Population Health Science Policy at Icahn School of Medicine at Mount Sinai, and Department of Epidemiology, Columbia University Mailman School of Public Health, New York, NY. The author can be reached at frances.howell28@gmail.com; **Micki Burdick** is an Assistant Professor in the Department of Women & Gender Studies at the University of Delaware, Newark, DE. Dr. Burdick can be reached at mburdick@udel.edu; **Oluwadamilola Oshewa** is a Clinical Research Coordinator in the Department of Obstetrics and Gynecology at the University of Pennsylvania, Philadelphia, PA. The author can be reached at Oluwadamilola.Oshewa@Pennmedicine.upenn.edu; **Maria Monterroso** is a Clinical Research Coordinator in the Department of Obstetrics and Gynecology at the University of Pennsylvania, Philadelphia, PA. The author can be reached at Maria.Monterroso@Pennmedicine.upenn.edu; **Alva Rodriguez** is a Clinical Research Coordinator in the Department of Health System Design and Global Health at Icahn School of Medicine at Mount Sinai, and Arnhold Institute for Global Health at Icahn School of Medicine at Mount Sinai, New York, NY. The author can be reached at Alva.RodriguezNunez@mssm.edu; **Camila Cabrera** is a Maternal Fetal Medicine Fellow in the Department of Obstetrics, Gynecology, and Reproductive Science at Icahn School of Medicine at Mount Sinai, New York, NY. Dr. Cabrera can be reached at camila.cabrera@mssm.edu; **Sheela Maru** is an Assistant Professor in the Department of Health System Design and Global Health at Icahn School of Medicine at Mount Sinai, and Arnhold Institute for Global Health at Icahn School of Medicine at Mount Sinai, New York City Health + Hospitals/Elmhurst, New York, NY. Dr. Maru can be reached at sheela.maru@mssm.edu; **Jennifer Lewey** is an Assistant Professor of Medicine in the Division of Cardiology at the University of Pennsylvania Perelman School of Medicine, Philadelphia, PA. Dr. Lewey can be reached at Jennifer.lewey@pennmedicine.upenn.edu; **Elizabeth A. Howell** is a Professor, Chair in the Department of Obstetrics and Gynecology, University of Pennsylvania, Philadelphia, PA. Dr. Howell can be reached at elizabeth.howell1@pennmedicine.upenn.edu; **Lisa Levine** is an Associate Professor, Chief, Maternal Fetal Medicine Division in the Department of Obstetrics and Gynecology at the University of Pennsylvania, Philadelphia, PA. Dr. Levine can be reached at lisa.levine@pennmedicine.upenn.edu; **Teresa Janevic** is an Associate Professor in the Department of Epidemiology at Columbia University Mailman School of Public Health, New York, NY. Dr. Janevic can be reached at tmj2101@cumc.columbia.edu

**Keywords:** Blood pressure, Hispanic or Latino, Language, Postpartum period, Text messaging

## Abstract

**Purpose:**

Research on remote monitoring for postpartum patients is lacking, particularly in a Spanish speaking population. We examined satisfaction and engagement with remote blood pressure monitoring by preferred language and other participant characteristics.

**Study Design and Methods:**

This was an observational longitudinal ohort study of *n* = 388 Asian, Black, and Hispanic postpartum patients from four hospitals from Philadelphia and New York City recruited between 2022 and 2023. English and Spanish speaking patients were enrolled. Participants were asked to track their blood pressures for 12 weeks after birth via a two-way text platform. We examined engagement with the platform (≥70% response to text prompts).

**Results:**

Most participants reported satisfaction with the program, with 92.0% of English speakers and 96.4% of Spanish speakers reporting satisfaction. Spanish speakers were more likely to engage in the program than English speakers (risk ratio: 1.22; 95% CI: 1.03, 1.44; adjusted risk ratio: 1.21; 95% CI: 1.01, 1.46).

**Clinical Implications:**

Among Spanish speakers, text-message-based remote blood pressure monitoring during the postpartum period was satisfactory. Spanish speakers were just as, if not more, likely than English speakers to engage in the remote monitoring program, suggesting the potential utility of remote monitoring for postpartum follow-up among a population at increased risk of adverse maternal outcomes.

## Abstract 2

Research on remote monitoring for postpartum patients is lacking, particularly in a Spanish speaking population. Satisfaction and engagement with remote blood pressure monitoring was evaluated in this study of 388 Asian, Black, and Hispanic postpartum patients from four hospitals from Philadelphia and New York City. Spanish speakers were just as, if not more, likely than English speakers to engage in the remote monitoring program, suggesting the potential utility of remote monitoring for postpartum follow-up among this population.

Supplemental Digital Content is available in the text

**Figure:**
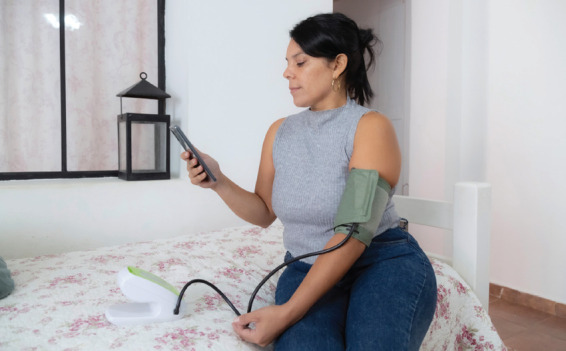


The COVID-19 pandemic accelerated the adoption of telehealth, telemedicine, and remote monitoring to assist in managing patients' health. However, there are concerns that with the expansion and perceived accessibility of telehealth, inequalities in access and uptake of telehealth and telemonitoring may still exist (Eberly et al., 2022). Remote monitoring allows for safe and effective monitoring from home, with providers responding in real-time if needed. Research on engagement and satisfaction with remote monitoring in patients for whom English is a second language (ESL) is lacking, particularly in the obstetric population.

Although the number of births to mothers of limited English proficiency is unknown, we know that in some regions, immigrant women of Hispanic origin are at an increased risk of poor maternal outcomes (Berger & Peled, 2022; Howell et al., 2017; Howell et al., 2020). Language discordance, or a lack of shared language proficiency between health care professional and patient, has been documented as a source of difficulty on the provider and patient end of communication (Nguyen et al., 2024). There is an urgent need to address this issue with innovative, flexible ways to monitor and manage patients' health during the postpartum period, particularly for historically underserved individuals.

Remote monitoring is increasingly used in pregnant and postpartum populations to manage and monitor blood pressure (BP). According to the World Health Organization (WHO) and the Centers for Disease Control and Prevention (CDC), pregnancy-related high BP (pre-eclampsia and eclampsia) remains a leading cause of maternal morbidity and mortality in the United States. In the United States, 3% of pregnancy-related deaths, which could occur during or 1 year after pregnancy, are due to hypertensive disorders of pregnancy (CDC, 2023). In 2019, The American College of Obstetricians and Gynecologists (ACOG) and the American Heart Association released guidance on cardiovascular risk following gestational hypertension and preeclampsia (ACOG, 2020). They recommended screening, diagnosis, and management of BP for pregnant women from prenatal to postpartum. They also recommend referral to a primary care provider or cardiologist to continue screening and management during the postpartum period (Mosca et al., 2011). ACOG recommends using telemedicine or remote health to screen, monitor, and care for people during the postpartum period (ACOG, 2018).

There is a dearth of research evaluating the satisfaction and engagement with a remote monitoring program for postpartum patients, particularly in a Spanish-speaking population. Language-concordant care (where there is a shared language proficiency between provider and patient or language access services are available) is urgently needed, as discordance leads to poor satisfaction with care and poorer health outcomes (Berger & Peled, 2022). This study is innovative in its evaluation of satisfaction with a remote BP monitoring platform using bilingual translated, automated text messages. Our objective was to measure associations between preferred language and level of engagement with a postpartum bi-directional text-based hypertension monitoring program. Our secondary objective was to describe satisfaction and ease of use of a remote text-based postpartum monitoring program in a multiethnic cohort and among Spanish-speaking postpartum women and birthing people.

## Methods

### Study Design

Participants in this study consented to participate in the coronaVirus Impact on Birth Equity in the 4th Trimester (VIBE) 2.0 Study, an observational longitudinal cohort study of *n* = 419 Asian, Black, and Hispanic postpartum patients from four hospitals from Philadelphia and New York City recruited between March 2022 and March 2023. Non-Hispanic White postpartum patients were not included in this study. Further details on the main study outcomes, recruitment, and enrollment can be found elsewhere (Janevic et al., 2025). Briefly, postpartum patients were invited to participate in this 3-month remote BP monitoring program during their birth hospital stay before discharge. Patients were approached, at their bedside, by trained, bilingual research coordinators who confirmed they met the eligibility criteria of self-identifying as Asian, Black, and/or Hispanic, spoke Spanish or English, were ≥18 years of age, and had a working cell phone. Baseline survey measures were collected at this time. We used an evidence-based, bidirectional SMS text-messaging platform developed by the University of Pennsylvania called Way To Health that delivers automated prompts to participants to submit BP readings and offers secure data capture for the study (Asch et al., 2012; Asch & Volpp, 2012). Way to Health also supported a clinically derived algorithm that responded automatically to out-of-range BP readings from participants with prompts for subsequent readings and directives in cases of emergent need. This automated algorithm has been successfully implemented by two of the included recruitment sites as standard of care for high-risk postpartum patients (Lopes Perdigao et al., 2021; Triebwasser et al., 2020). Study clinicians at each site monitored submitted readings daily. Prior to the study's start, text messages through Way to Health were written in English. Text messages were translated to Spanish by a native Spanish speaker on our team and tested by other bilingual team members. Participants were asked to complete a survey at baseline and at 3-month follow-up administered using the University of Pennsylvania's REDCap. A monetary incentive ($25) was provided following the completion of each survey via gift card. Institutional Review Board approval was obtained from the University of Pennsylvania Human Research Protections Program, the Icahn School of Medicine Program for the Protection of Human Subjects, and the NYC Health and Hospitals Elmhurst and Queens Research Committee.

Upon consent and enrollment in the study, participants completed a baseline survey and were instructed on proper use of the BP cuff and text-messaging program. They were prompted to submit two BP measures per day for the first 10 days of their enrollment in the study, followed by two BP measures per week for the remaining 12 weeks of their enrollment in the study (43 BP measure prompts in total).

### Measures

#### Measure of Engagement

Based on the definition of engagement determined by the study team *a priori*, we defined adequate engagement level as those who responded to 70% or more of the text prompts, whereas less than 70% was considered a “low” level of engagement (Parati et al., 2010; Price et al., 2014; Thompson et al., 2023). Participants had a window of time to respond to a text prompt before the response was no longer counted as a response to that particular prompt, but texts that were sent outside of each response window were counted toward engagement in this analysis.

#### Validated Survey Measures Collected at Baseline

Survey measures were collected via an electronically delivered survey.

Sociodemographic characteristics were collected during the baseline survey and were self-reported. These characteristics included age (younger than 30 vs. 30 or older), relationship status (married, living with a partner, or in a relationship vs. not married, living with a partner, or in a relationship), education (high school/equivalent or less vs. some college or higher), race/ethnicity, and nativity (US-born vs. foreign-born).

Satisfaction items were based on validated instruments from Eberly et al. (2022), Hirshberg et al. (2018), and Bracken et al. (2014). Satisfaction was measured on a Likert scale from unsatisfied to very satisfied, dichotomized to “satisfied” and “not satisfied” categories for analysis. Ease of use of the text-messaging platform was measured with a Likert scale with a neutral response option. We also inquired about barriers to engagement with the program, with an option for participants to provide open-text responses.

Language: We defined language selection in this study by the language in which the participant selected to receive their text prompts for BP submission (English or Spanish).

We selected covariates we theorized might be associated with our study outcomes.

Depression: We examined the presence of depression at baseline using the Edinburgh Postnatal Depression Scale, which is a 10-item scale used clinically as a screening tool to detect presence of depression during the postpartum period (Cox et al., 1987). We used a median split method to define those with and without depressive symptoms, where participants with a score of 11 or higher were considered to have median or above depressive symptoms.

Anxiety: Anxiety was measured with the Generalized Anxiety Disorder scale, a 7-item Likert scale (Spitzer et al., 2006). We dichotomized results into those who had none or mild anxiety (<10) and those who had moderate to severe anxiety (≥10) based on the suggested scoring criteria by Spitzer et al. (2006). For both measures, we used single mean imputation to prevent missing data among participants who had completed 2/3 or more of the total scale items. Final binary measures were computed using imputed values.

Obstetric experiences: We measured aspects of the obstetric care experience, including gendered racial microaggressions in obstetric encounters and satisfaction with the childbirth experience. Gendered racial microaggressions are insidious put-downs and discriminatory practices connected to both race and gender (Lewis & Neville, 2015). As the original scale was designed for Black women only and was not designed explicitly for obstetric care, we garnered expertise from our community advisory board to modify the Gendered Racial Microaggressions Scale (GRMS) to apply to the study population and within pregnancy, birth, and postpartum care. The modified version of the scale was found to be valid in the Asian, Black, and Hispanic population, as well as a valid tool for capturing gendered racial microaggressions in the obstetric setting for those that speak Spanish (Howell et al., 2024). We analyzed GRMS as a binary measure and captured whether the participant had experienced any instances of gendered racial microaggressions during their obstetric care (e.g., I was told to calm down). Participants' experiences during their birth were measured with the Birth Satisfaction Scale Revised (BSS-R) version (Hollins Martin & Martin, 2014). Birth experiences are measured with 10 items within three domains: quality of care provision, the patients' personal attributes, and stress experienced during labor. The BSS-R scale was binarized using a median split approach.

#### Electronic Medical Record Data

The following clinical measures were ascertained from the electronic medical record: mode of birth (vaginal vs. cesarean section), age at index birth, parity (nulliparous vs. multiparous), and insurance (private, public, and unknown). Adequate prenatal care was measured using the Kotelchuck index, which accounts for the timing of initiation into prenatal care and the number of total visits, adjusted for gestational age at birth and categorizes prenatal care utilization as inadequate, intermediate, adequate, or intensive (Kotelchuck, 1994). Adequate prenatal care was included as a proxy for evidence of prior degree of engagement in care during pregnancy.

Cardiovascular risk level (high-risk or not high-risk) was determined by presence of a hypertensive disorder, including chronic hypertension, gestational hypertension, and preeclampsia and was ascertained via electronic medical record.

### Statistical Analysis

We first described participant sociodemographic characteristics by language preference and generated summary statistics of satisfaction with the program (frequency, proportions). In bivariate analyses, we tested the association between each sociodemographic and birth characteristic with language selection, using Chi-square tests and two-sided t-tests. We then examined all sociodemographic and birth characteristics by engagement level with the text-messaging program.

We performed log binomial regression to examine the association between prenatal and immediate postpartum characteristics and level of engagement (low vs. adequate engagement) with the text-messaging program. We used a complete case approach in which cases with missing data on key variables (education) were excluded from analyses, leaving *n* = 388 cases (out of *N* = 419) in the final model. We chose potential confounders based on a Directed Acyclic Graph (age, nativity, and education) and estimated covariate-adjusted models.

### Sensitivity Analyses

Given this, we conducted a sensitivity analysis excluding participants who reported receiving some prenatal care outside of study institutions (*n* = 78). We also conducted a second sensitivity analysis including hospital of recruitment in the model to control for site-level differences, such as quality of care, which may influence level of engagement in ongoing BP monitoring. We conducted a sensitivity analysis altering our definition of engagement as response to 80% or more text prompts. Data management and analyses were conducted using SAS v. 9.4 and SPSS, v. 29.

## Results

Of the *N* = 419 participants enrolled in the study, *n* = 31 had missing data on key variables and were excluded from this analysis. Characteristics of the *n* = 388 VIBE 2.0 study participants with complete data are presented in Table 1 and Supplementary Table 1 (Supplemental Digital Content at http://links.lww.com/MCN/A108). Participants' ages ranged from 16 to 46, with a mean age of 30.0 years. Most participants (*n* = 139; 35.8%) achieved a high school level of education or received a GED, whereas *n* = 93 attended some college (24.0%) or beyond (*n* = 95; 24.5%). Most participants reported they were married or living with a partner (*n* = 280; 72.2%).

Most participants chose English as their preferred language for text messaging through the Way to Health platform (*n* = 289; 74.5%). In bivariate analyses, participant characteristics found to be associated with language preference included marital status, education level, nativity, year of migration, race/ethnicity, and cardiovascular risk level (Supplementary Table 1 at http://links.lww.com/MCN/A108). Participant characteristics associated with the level of engagement, presented in Table 1, included increasing age, being married or living with a partner, higher education level, being foreign-born, and race and ethnicity.

**TABLE 1.** Participant Characteristics by Level of Engagement

Variables	Overall Cohort	Low Engagement	Adequate Engagement	*P*-value
	*N*=388	*n*=175 (45.1%)	*n*=213 (54.9%)	
	*n* (%)	*n* (%)	*n* (%)	
Age at Birth				
<20 years	12 (3.1)	10 (83.3)	2 (16.7)	<.001
20-29 years	168 (43.3)	96 (57.1)	72 (42.9)	
30-34 years	117 (30.2)	45 (38.5)	72 (42.9)	
35-39 years	71 (18.3)	21 (29.6)	50 (70.4)	
40 years and up	20 (5.1)	3 (15.0)	17 (85.0)	
Marital Status				.019
Married or living with partner	280 (72.2)	116 (41.4)	164 (58.6)	
Not married or living with partner	108 (27.8)	59 (54.6)	49 (45.4)	
Education Level				.008
Less than HS	61 (15.7)	29 (47.5)	32 (52.5)	
HS or GED	139 (35.8)	77 (55.4)	62 (44.6)	
Some college	93 (24.0)	36 (38.7)	57 (61.3)	
BA or higher	95 (24.5)	33 (34.7)	62 (65.3)	
Country of Birth				.026
U.S. born	182 (46.9)	93 (51.1)	89 (48.9)	
Foreign-born	206 (53.1)	82 (39.8)	124 (60.2)	
Year of Migration (*n*=196)				.692
Within last 5 yr	97 (49.5)	41 (42.3)	56 (57.7)	
5-10 yr ago	37 (18.9)	15 (40.5)	22 (59.5)	
>10 yr ago	62 (31.6)	22 (35.5)	40 (64.5)	
Race or Ethnicity				.020
Black (non-Hispanic)	155 (39.9)	84 (54.2)	71 (45.8)	
Hispanic or Latina	149 (38.4)	62 (41.6)	87 (58.4)	
Asian (non-Hispanic)	40 (10.3)	13 (32.5)	27 (67.5)	
Other or Multiple Races	44 (11.3)	16 (36.4)	28 (63.6)	
Adequacy of Prenatal Care (Kotelchuck Index)				<.001
Adequate or intensive	83 (21.4)	23 (27.7)	60 (72.3)	
Intermediate or inadequate	268 (69.1)	129 (48.1)	139 (51.9)	
Missing or no prenatal care	37 (9.5)	23 (62.2)	14 (37.8)	
Mode of Birth				.014
Vaginal	268 (69.1)	132 (49.3)	136 (50.7)	
Cesarean	120 (30.9)	43 (35.8)	77 (64.2)	
Nulliparous				.245
No	271 (69.8)	117 (43.2)	154 (56.8)	
Yes	117 (30.2)	58 (49.6)	59 (50.4)	
Maternity Leave				.422
Took any amount of leave	91 (16.2)	25 (27.5)	66 (72.5)	
No leave	29 (7.6)	12 (41.4)	17 (58.6)	
Stay at home parent or not looking for work	107 (27.4)	38 (35.5)	69 (64.5)	
Unemployed or looking for work	48 (12.2)	18 (37.5)	30 (62.5)	
EPDS				.849
Depressed	52 (13.9)	23 (38.5)	29 (61.5)	
Not depressed	322 (86.1)	147 (45.7)	175 (54.3)	
GAD-7				.931
None or mild anxiety	347 (91.3)	155 (44.7)	1912 (55.3)	
Moderate to severe anxiety	33 (8.7)	15 (45.5)	18 (54.5)	
Language Selection				.024
English	289 (74.5)	140 (48.4)	149 (51.6)	
Spanish	99 (25.5)	35 (35.4)	64 (64.6)	
Risk Level				.656
Low	266 (68.6)	122 (45.9)	144 (54.1)	
High	122 (31.4)	53 (43.4)	69 (56.6)	

*Note.* High risk was defined as presence of a pre-existing or pregnancy-related hypertensive disorder.

HS: High school; GED: General Educational Development or High School Equivalency Test.

EPDS: Edinburgh Postnatal Depression Scale; GAD-7: Generalized Anxiety Disorder scale.

Risk ratios reporting associations between language selection and level of engagement with the text-messaging program can be found within text, here. The final model was adjusted by the following variables: education, marital status, nativity, and adequacy of prenatal care. Spanish-speaking participants (*n* = 100; 25.8%) were more likely to engage with the text-messaging platform than those that were English speakers (68.7% vs. 56.4%, risk ratio [RR]: 1.22; 95% CI: 1.03, 1.44; adjusted risk ratio [aRR]: 1.21; 95% CI: 1.01, 1.46). The sensitivity analysis, which included hospital recruitment in the model to control for site-level differences, had similar results (aRR = 1.16, 95% CI = 0.93, 1.45). The results were also similar in the sensitivity analysis in which we made the definition for “engagement” stricter: aRR = 1.22, 95% CI = 0.99, 1.49.

Results of our exploratory analysis of satisfaction and ease of use survey items can be found in Table 2. Of the 71.9% (*n* = 279) of the total sample that completed the follow-up survey, there was no significant difference in the level of satisfaction or reported ease of use by language selection (English or Spanish) in this cohort with high rates of both satisfaction and ease of use in both groups. Most participants reported satisfaction with the program, with 92.6% of English speakers and 96.1% of Spanish speakers reporting satisfaction with the text-messaging program. Reporting being busy with children or other activities were the most frequently selected barriers to responding to BP prompts. Free text responses revealed that many participants were out of the home when they received a BP prompt and forgot to send a response upon returning home. Others mentioned the timing of the text messages felt inconsistent, and they would have preferred texts to be sent on predictable days and times of the week.

**TABLE 2.** Satisfaction and Ease of Use of Text-Messaging Platform by Language Selection

Satisfaction	English *n* = 202 (72.4%)	Spanish *n* = 77 (27.6%)
	*n* (%)	*n* (%)
How satisfied are you with the text-messaging reminders?		
Satisfied	187 (92.6)	74 (96.1)
Not satisfied	15 (7.4)	3 (3.9)
How easy was it to respond with your blood pressure and weight?		
Easy	183 (90.1)	70 (92.1)
Neither easy nor difficult	14 (6.9)	2 (2.6)
Difficult	6 (3.0)	4 (5.3)
Barriers to responding to text messages		
I sent in a blood pressure with every reminder	65 (68.4)	30 (31.6)
I was too busy taking care of baby or other kids	114 (75.5)	37 (32.2)
I have too many other responsibilities	42 (84.0)	8 (16.0)
I was worried about additional charges from cell phone carrier	1 (100.0)	0 (0.0)
My partner did not want me to respond	0	0
I didn't think it was important	2 (66.7)	1 (33.3)
Other	27 (75.0)	9 (25.0)

*Note.* Barriers were asked with a “select all that apply” option, and a participant may have reported more than one barrier. Percentages will not add to 100%.

## Discussion

Our study of postpartum Asian, Black, and Hispanic patients sheds light on participant engagement and satisfaction with a remote BP monitoring program, focused on patients whose preferred language is Spanish. Our study aimed to assess the influence of language preference on engagement in participants, and whether their level of engagement and satisfaction differed by their preferred language. We found that higher education and Spanish language preference were associated with a higher level of engagement in the remote monitoring program. Including a significant proportion of Spanish-speaking participants in the VIBE 2.0 Study is a strength and allowed for a nuanced exploration of language-specific engagement and satisfaction. Patients with limited English proficiency are at higher risk for poor health outcomes than patients whose first language is English, likely due to inadequate resources both within and outside the health care system that addresses communication barriers (Twersky et al., 2024). Persons with limited English proficiency have historically demonstrated lower participation in mobile health technologies (Balakrishnan et al., 2022). Our study demonstrates that among Spanish speakers, a text-message-based remote BP monitoring program during the postpartum period was both satisfactory and easy to use. In our study, Spanish-speaking participants were more likely to engage with the automated text-messaging program than English-speaking participants (Machen et al., 2021), underscoring the importance of language inclusivity in remote technology interventions, as language is a key component in care quality.

Our study also provided valuable descriptive information on engagement in remote BP monitoring, using a wide range of covariates not commonly available. We found that participants with a high school or GED education level were less likely to engage in remote BP monitoring than those with higher education levels. This is consistent with literature suggesting that higher education is associated with increased use of, experience with, and adherence to eHealth programs (Simblett et al., 2018). Given the strong intercorrelation of education and income, this association may also be influenced by income and access to technological resources, leading to higher eHealth literacy. Our experience also supports this finding: we anecdotally noted in our recruitment tracking that several participants dropped out of the study because they lost access to their phone plans or devices. Future research and design of eHealth interventions should account for potential income and educational disparities in access and use of the platform. Contrary to previous studies, we did not find that people with hypertensive disorders of pregnancy had a higher level of engagement compared to low-risk (Hellem et al., 2023). It is unclear why we did not find this association, although it is possible that the monetary incentive provided in this study increased engagement with the program in both high- and low-risk participants.

### Limitations and Strengths

Although our study has many strengths, we recognize that our results should be interpreted within the context of limitations noted herein. The measurement of participant's level of engagement used in our study accounts for the overall frequency of messages received and does not account for the number of prompts returned or not returned. This could underestimate the number of text messages sent in by participants and bias our results toward the null, that there is no association between participant characteristics and level of engagement with the Way to Health platform. Another limitation was the smaller sample size of participants whose preferred language was Spanish, which limited our ability to further examine characteristics within Spanish speakers that were associated with higher/lower engagement. Future research should oversample for Spanish speaking participants so that these characteristics could be meaningfully studied. Our study's strengths include the inclusion of a multiethnic cohort of Asian, Black, and Hispanic participants and the wide array of survey variables available for analysis.

## Clinical Implications

Our study provides important insight into remote monitoring interventions for multilingual populations. Many English-only remote monitoring applications exclude the populations that may benefit from closer monitoring in the postpartum period. As more health care institutions are using remote platforms in maternal health settings, it is paramount that health care leaders select programs that have been user tested in multilingual populations. Our study demonstrated that the Way to Health text-messaging intervention was found to be user-friendly and satisfactory. Nurses are often confronted with challenges connecting with and engaging patients with limited English proficiency, and it is worth noting that even in-hospital care (where interpretive resources are readily available) can be inadequate and delayed for these patients. Appropriate translation and testing of remote text-messaging applications can bridge the gap in care between patients whose first language is English and patients with limited English proficiency. Hospital and clinic administrators should consider the needs of their patient population when choosing remote monitoring software.

### Acknowledgment

Research reported in this publication was supported by the National Institutes of Health (NIH) National Institutes on Minority Health and Health Disparities (NIMHD) under award 1R01MD016029-02S; PI: Elizabeth Howell.

## CLINICAL IMPLICATIONS

- Hospital clinical leaders selecting programs using text-messaging software should choose remote monitoring programs that have been tested in multiple languages.
- Nurses should assess patients for their preferred language of care provider and text messages to ensure language concordance.
- Patients with less than a college education and those with inadequate prenatal care may be less likely to engage in remote blood pressure monitoring. These patients may benefit from interventions providing additional nursing support and education about the rationale for remote blood pressure monitoring and support engaging in general with their health care.
- Nurses should recognize that patients with limited English proficiency may have difficulties with consistent access to mobile phones and may leave the country after their birth. Postpartum care plans should be discussed with these patients considering their plans for travel and access to a cellular phone.
- Access to remote monitoring that is language concordant may contribute to postpartum health equity.

## Congratulations to MCN 2025 Articles of the Year Awards

**Each year, the MCN editorial board votes on the best articles of the year awards in 3 categories. This year there was a tie for the Scholarly Review Article of the Year, so there 2 winners in this category**

2025 Scholarly Review Article of the Year

**Nurses' Role in the Birth Experience: An Integrative Review**

Nicholas M. Raposo, MPH, BSN, RNC-OB & Corrine Y. Jurgens, PhD, RN, ANP, FAHA, FHFSA, FAAN

Published in the March / April 2025 issue of MCN

2025 Scholarly Review Article of the Year

**Labor and Delivery Nurse Psychological Trauma: An Integrative Review**

Maggie C. Runyon, MSN, RNC-OB; Kimberly K. Trout, PhD, RN, CNM, FACNM, FAAN; Linda Carman Copel, PhD, RN, PMHCNS, BC, CNE, ANEF, NCC, CGP, FAPA; & Helene Moriarty, PhD, RN, FAAN

Published in the September / October 2025 issue of MCN

2025 Quality Improvement Article of the Year

**Keeping Mothers Together with Their Babies Requiring Neonatal Intensive Care During the Birth Hospitalization: An Innovative Model of Care**

Amy Dagestad, MBA, MSN, RN, NE-BC, RNC-OB, FAWHONN

Published in the May / June 2025 issue of MCN

2025 Practice Article of the Year

**Collaborative Approaches in the Emergency Department for Maternity Patients**

Miriam Wright, MSN, RNC-OB, CPHQ, C-ONQS & Tracey Jones, MSN, RNC-OB, C-EFM, C-ONQS

Published in the November / December 2025 issue of MCN
